# Bolstering the willingness to uptake covid-19 vaccination through multidisciplinary health communication intervention: a cue for reaching herd immunity in Nigeria

**DOI:** 10.4314/ahs.v23i4.19

**Published:** 2023-12

**Authors:** Ndidi C Ibenyenwa, Ogechi K Onyekwere, Ndubuisi F Ugwu, Adijat B Adams, Philip I Ajewole, Veronica I Makinde, Blessing N Onyekachi, Anselm U Anibueze, J K Opele, Onyemaechi F Nwogu

**Affiliations:** 1 Department of Mass Communication, Federal University Oye-Ekiti, Nigeria; 2 Department of Physical and Health Education, Federal College of Education, Eha-Amufu, Enugu State, Nigeria; 3 Department of Health Education, Federal University Oye-Ekiti, Nigeria; 4 Department of Guidance and Counseling, Federal University Oye-Ekiti, Nigeria; 5 Department of Educational Management and Business Studies, Federal University, Oye-Ekiti, Nigeria; 6 Department of Counseling Psychology, Bamidele Olumilua University of Edu., Sc, & Tech., Ikere-Ekiti, Nigeria; 7 Department of Psychology, University of Nigeria, Nsukka; 8 Department of Library and Information Science, Federal University Oye-Ekiti, Nigeria

**Keywords:** Herd immunity, Nigeria, Vaccination, Health Communication intervention, Unvaccinated community dwellers

## Abstract

**Background:**

The prevailing unwillingness to accept COVID-19 vaccination among the eligible population has been a serious setback in Nigeria's bid to reach herd immunity against the pandemic.

**Objective:**

We assessed the impact of a multidisciplinary health communication intervention (MHCI) on willingness to accept COVID-19 vaccination among eligible unvaccinated community dwellers (EUCD) in Nsukka Urban, Enugu State, Nigeria.

**Methods:**

Through a quasi-experiment that adopted a pre-test, post-test, non-control group design, we studied a total of 85 eligible unvaccinated adults. A researcher-designed “Willingness to Accept COVID-19 Vaccination Questionnaire” was the instrument used for data collection. The data gathered was analysed by means of Statistical Package for Social Sciences (SPSS) version 23.0. Specifically, descriptive and inferential statistics were used to test the formulated research questions and the hypothesis at 0.05 level of significance.

**Results:**

We found that the mean scores of willingness to accept COVID-19 vaccination increased significantly after the treatment. There was no significant interaction effect of gender, level of education, and age on the mean of willingness to accept COVID-19 vaccination scores of EUCD after MHCI.

**Conclusion:**

The study established that MHCI is impactful in bolstering the willingness to accept COVID-19 vaccination. The Nigerian government should adopt and implement this intervention in schools, communities, and other institutions in order to attain herd immunity in Nigeria.

## Introduction

The novel Coronavirus disease of 2019 (COVID-19) has become a serious public health challenge worldwide. Reaching herd immunity has been suggested as an evidence-based option for keeping communities safe and protected from the virus.^1^, ^2^, ^3^ Herd-immunity (HI) is the type of protection achieved when substantial proportion of community members has developed immunity against an infectious disease by means of immunity acquired due to prior infection or through vaccination, in which the likelihood of the disease to spread in the community is diminished.^1^, ^4^ Although individuals who are exposed to the virus can develop immunity against it if they recover, adopting this pathway as a means of reaching HI is described as unethical, scientifically problematic, and will lead to unwarranted diseases, sufferings and deaths^3^. Against this backdrop, the World Health Organization (WHO) has recommended that vaccination is a healthier route for reaching HI against COVID-19. 3

Receiving COVID-19 vaccine would prompt one's immune system to produce disease-fighting proteins called antibodies through the use of innocuous version of S protein, a spike-like structure on the surface of the virus.^1^, ^2^ With this vaccine, the antibodies are capable of fighting the virus if one is exposed to it. COVID-19 vaccine has been proven to be sufficiently potent in preventing one from getting infected, becoming seriously ill or dying as a result of the virus. 3, 6, 7 Thus, vaccination of a critical mass of the global population has been shown to be crucial for halting the pandemic. 8 In view of this, a target of 70% vaccine coverage has been set by WHO before the mid-2022. 9 Pursuant to this, there has been a dramatic increase in the rate of vaccines uptake globally. Dozens of countries like United Arab Emirates, Singapore, and Qatar have had up to 75% of their population fully vaccinated. 8 The United Nations reports 9 shows that more than 10 billion doses have already been administered worldwide. Regrettably, billions of people remain unvaccinated globally and majority of them are from low-income countries. 8 Specifically, of more than 10 billion doses already given out worldwide, less than 10% happened in poor countries. 9, 10 With this trajectory, it would be difficult to reach herd immunity in poor countries including Nigeria.

In Nigeria, only 10% of the total eligible population targeted for COVID-19 vaccination had been fully vaccinated as of June, 2022, the period of this study. 11 This was way too low compared to at least 70% full vaccination targeted by June, 2022 if Nigeria must achieve herd immunity against the virus in expected time. 12 In a bid to up-scaling vaccination coverage, Nigerian Government established vaccination centres in different public places outside health facilities. 1 In spite of these efforts, uptake of COVID-19 vaccine remained very low. 7, 14

The poor vaccination coverage was blamed on the unwillingness of the eligible people to accept vaccination, a phenomenon known as vaccine hesitancy. 7 Since its outbreak, COVID-19 has been chockfull with infodemics arising from conspiracy theories, hoaxes, damaging stereotypes, bogus myths, and stigmatization. 15, 16 These have created fears, doubts, disbelief, uncertainties and concerns about its safety and side effects making most Nigerians skeptical and unwilling to accept its vaccine. 15, 17, 18, 19 With these mindsets, reaching herd immunity will continue to elude Nigeria unless there is an intervention to build vaccine confidence among eligible Nigerians. Regrettably, researchers have not devoted much needed attention in disabusing Nigerians of these erroneous beliefs and fears which are responsible for this negative attitude to the vaccine. Therefore, the present study was an attempt to promote the willingness to accept COVID-19 vaccine among eligible unvaccinated community dwellers (EUCDs) through a Multidisciplinary Health Communication Intervention (MHCI). Specifically, the study examined the impact of MHCI on: (i) willingness to accept the initial or primary COVID-19 vaccination; (ii) willingness to accept booster doses; and (iii) willingness to encourage others to get vaccinated.

## Methods

### Study design and participants

We conducted a quasi-experimental study that adopted pretest post-test non-equivalent comparison (control) group design. The study participants were all the 217 community dwellers who attended COVID-19 enlightenment programme held between 12^th^ to 14^th^ April 2022, at government's field of Nsukka Local Government Area of Enugu State, Nigeria. Purposive sampling method was used in selecting 85 unvaccinated participants who are 18 years and above, who indicated readiness and have given consent to participate fully in the study.

### Data collection

A researcher-designed “Willingness to Accept COVID-19 Vaccination Questionnaire (WACVQ)” was the instrument used for data collection. The WACVQ consisted of two sections, namely: A and B. Section A was to elicit information on participants' demographic characteristics including: age, gender and level of educational. Section B had 22 items which examined the participants' willingness to accept COVID-19 Vaccine. The participants were to indicate whether they were: “Very Willing (VW)”, “Willing (W)”, “Unwilling (U)”, or “Very Unwilling (VU)” in the items that were provided. The WACVQ was validated by a total of five experts. Reliability test that yielded coefficient of 0.78 was conducted on the instrument using split-half method (Spearman Brown Coefficient).

### Procedures

After obtainment of permission from the Nsukka Local Government health authorities and the Nsukka Town Union president, the researchers placed a one-month advertisements for COVID-19 enlightenment programme for community members using radio jingles, fliers and town criers across the community between March and April, 2022. A total of 219 people attended the programme from which 85 EUCDs were purposively selected for the study. The mode of delivery of this empowerment programme was through physical meeting of the researchers and the participants. COVID-19 prevention protocols were strictly observed throughout the programme.

Before the commencement of the treatment, the WACVQ was administered to the subjects in order to secure their pre-test scores. To ensure full participation, attendance slip was designed to cover the three-day programme through which the participants were signed in and out for the three days. In order to get the post-test scores, the items in the WACVQ were randomly rearranged and re-administered to the participants after the treatment. The participants were served with snacks and drinks. They were promised that they would be given souvenir of T-shirt and COVID-19 protection materials after the training. All of these were to motivate their interest to participate.

This multidisciplinary intervention was presented to the participants by the present researchers who are experts with PhD in selected disciplines including: Public Health Education, Psychology, Health Information Management and Biostatistics, Educational Management, and Counseling. Other experts were from Mass Communication, Sociology and Library and Information Science. Four research assistants who were postgraduate students of the University of Nigeria, Nsukka were employed to assist in the study.

### Intervention

The Multidisciplinary Health Communication Intervention manual was used for the programme. The manual was designed with cues from Knowles's Adult Learning Theory (Androgogy)^20^ and principles of effective health communication. 21, 22 Topics that formed the contents of the manual were taken from fields of Public Health Education, Health Information Management and Biostatistics, Sociology, Psychology, Mass Communication, Counseling, library and information science. The intervention had three segments covering cognitive, affective and psychomotor domains. The cognitive segment focused on sharing knowledge and developing in the participants the intellectual skill relating to COVID19. In this segment, pieces of scientific information about COVID-19 including its origin, nature, communicability, prevention and vaccination were given. The affective segment focused on building positive attitudes in the subjects by disabusing erroneous beliefs, myths and conspiracy theories relating to COVID-19. The psychomotor segment was directed towards instructing the participants on practical steps to help prevent them from contacting and/or spreading the virus. Emphasis was placed on vaccination, and the reason for the mild side effects of vaccination was scientifically clarified. The three segments were presented in three days respectively. In all, the participants were taught to do away with myths, erroneous beliefs and conspiracy theories. They were also made to understand, with pieces of evidence, that COVID-19 really exists and that vaccination remains a sure way of dealing with it.

### Ethical approval

In addition to securing approval for this study from the Research and Ethic Committee of the Department of Human Kinetics and Health Education, Federal University Oye-Ekiti, Nigeria (Ref: HKHE/2022/REC/014-R2), the present researchers satisfied the Helsinki Declaration on ethical principles for research involving human subjects 23 and the ethical principles of American Psychological Association. 24

### Data analysis

Analysis of data emanating from the present study was done by means of IBM Statistical Package for Social Sciences (SPSS) version 23.0. Frequencies and percentages were used to describe the subjects' demographic characteristics (gender, age and level of education). Mean and Standard Deviation were used in describing the willingness to accept COVID-19 vaccine before and after the intervention. Analysis of Covariance (ANCOVA) was used to examine the interaction effects of gender, age and level of education on the participants' willingness to accept COVID-19 vaccine. All the tests were 2-tailed, and the probability values less than 0.05 (p<0.05) were considered significant.

## Results

Table 1 shows that of the 85 respondents, 36 (42.4%) were Males while 49 (57.6%) were females. A total of 6 (7.1%) respondents had no formal education, 15 (17.6%) of the respondents had Primary/O' level certificate, 34 (10.0%) had OND/NCE certificate, 18 (21.2%) had HND/bachelor's degree, while 12 (14.1%) had Postgraduate degree. In terms of age, 29(34.1%) were younger adult and 44(51.8%) were middle age adults while 12(14.1%) were older adults.

**Table 1:** Demographic Characteristics of Respondents (N= 85)

Demographic Details	Frequency (f)	Percentages (%)
**Gender**		
Male	36	42.4
Female	49	57.6
**Level of Education**		
No Formal Education	6	7.1
SSCE or below	15	17.6
OND/NCE	34	40.0
Bachelor Degree	18	21.2
Postgraduate Degree	12	14.1
**Age Group**		
18-35 years (younger adults)	29	34.1
36-55 years (middle age adults)	44	51.8
56 years or more (older adult)	12	14.1

Table 2 shows that the willingness to accept COVID-19 vaccination rose after the HCI. The grand mean scores of the willingness to accept initial doses of COVID-19 vaccination; the willingness to accept booster doses; and the willingness to encourage others increased significantly from pre-test (M=1.80, SD=0.75) to post-test (M=3.04, SD=0.66); pre-test (M=1.75, SD=0.63) to post-test (M=2.85, SD=0.58); and pre-test (M=1.71, SD=0.69) to post-test (M=3.14, SD=0.63) respectively.

**Table 2:** Mean Scores of willingness to Accept COVID-19 Vaccine (N= 85)

Measures		Pre-test	Post-test	Mean Gain
		x̅ (SD)	x̅ (SD)	x̅ (SD)
**Willingness to accept initial dose of COVID-19 vaccine**				
	1. Willing to get vaccinated not minding stories concerning its safety	2.02(0.71)	3.07 (0.63)	1.02 (0.07)
	2. Willing to get vaccinated in spite of its rumoured side effects stories	1.66(0.75)	2.93 (0.59)	1.27 (0.16)
	3. Willing to get vaccinated amidst fear of unknown	1.69(0.71)	3.13 (0.59)	1.44 (0.11)
	4. Willing to get vaccinated NOT because of official requirement	1.73(0.80)	3.72(0.45)	1.99 (0.34)
	5. Willing to accept vaccination notwithstanding cultural or religious barriers.	2.32(0.77)	3.31(0.60)	1.00(0.17)
	6. Willing to cancel an important appointment to get vaccinated	1.61(0.82)	2.54 (0.80)	0.93 (0.02)
	7. Willing to endure a long queue to get vaccinated	2.46(0.93)	3.12(0.70)	0.66 (0.24)
	8. Willing to spend money (if necessary) to get vaccinated	1.49(0.73)	2.58(0.79)	1.08 (0.06)
	9. Willing to travel distances to get vaccinated	1.24(0.48)	2.58(0.66)	1.34 (0.16)
	10. Willing to resist discouragement from my significant others to get vaccinated	1.79(0.80)	3.40(0.76)	1.61(0.04)
**Grand mean**		**1.80(0.75)**	**3.04 (0.66)**	**1.24(0.09)**
**Willingness to accept booster doses**				
	1. Willing to accept a booster dose even if I experienced some side effects in the initial shot	1.99(0.85)	3.02(0.64)	1.4 (0.40)
	2. Willing to accept a booster dose even if I have to pay	1.42(0.50)	2.91(0.72)	1.48 (0.76)
	3. Willing to accept a booster dose notwithstanding the distance	2.33(0.92)	3.05(0.49)	0.72 (0.23)
	4. Willing to accept a booster dose at the expense of my convenience	1.26(0.58)	2.74(0.53)	1.48 (0.94)
	5. Willing to get a booster dose NOT because of official requirement	1.86(0.35)	2.33(0.56)	0.47 (0.09)
	6. Willing to overcome scaremongering and get a booster dose	1.66(0.56)	3.02(0.56)	1.36(0.81)
**Grand mean**		**1.75 (0.63)**	**2.85 (0.58)**	**1.09 (0.51)**
**Willingness to encourage others to get vaccinated**				
	1. Willing to allow your relations to get vaccinated	2.28(0.77)	3.26(0.62)	0.98 (0.21)
	2. Willing to ensure that your family and friends are vaccinated	1.83(0.93)	3.19(0.66)	1.36 (0.44)
	3. Willing to support others financially (if necessary) to get vaccinated	1.40(0.58)	2.76(0.57)	1.36 (0.78
	4. Willing to convince others to get vaccinated	1.59(0.93)	3.21(0.74)	1.62 (0.69)
	5. Willing to encourage others to take booster doses	1.47(0.50)	3.09(0.57)	1.62 (1.12)
	6. Willing to painstakingly explain to others the need for the vaccine	1.72(0.45)	3.32(0.64)	1.60 (1.15)
**Grand mean**		**1.71(0.69)**	**3.14 (0.63)**	**1.43 (0.73)**

Table 3 shows that there is no significant interaction of gender (F = 0.738; P > 0.05), educational level (F =0.568, P< 0.05), and age (F =0.240, P> 0.05) on the mean scores of willingness to accept COVID-19 vaccination before and after the intervention.

**Table 3:** Interactive Effect of Education Level, Gender and Age on the Means Scores of Willingness to Accept COVID-19 Vaccine

Tests of between-subjects effects					
Dependent Variable: Treatment Group					
Source	Type III Sum of Squares	df	Mean Square	F	Sig.
Corrected Model	5.908a	34	.174	.641	.934
Intercept	195.632	1	195.632	721.753	.000
Gender	.200	1	.200	.738	.392
Age	.260	4	.065	.240	.915
Education	.616	4	.154	.568	.686
Gender * Age	.571	4	.143	.526	.716
Gender * Education	.391	4	.098	.361	.836
Age * Education	2.431	13	.187	.690	.770
Gender * Age * Education	1.971	4	.493	1.818	.129
Error	36.592	135	.271		
Total	425.000	170			
Corrected Total	42.500	169			

***a. R Squared = .139 (Adjusted R Squared = .078)***

## Discussion

Findings from this study reveal that the MHCI significantly bolstered the willingness to accept initial and booster doses, as well as the willingness to encourage relations to accept COVID-19 vaccination. The improved willingness after MHCI is not surprising and can be attributed to the employment of evidence-based health education, as well as principles of counseling and effective health communication in the planning and implementation of the intervention. It has earlier been documented that comprehensive health promotion programmes are solutions for bolstering positive healthy behaviour. 25 For instance, an evidence-based health education and communication was coordinated to enhance COVID-19 vaccine acceptance across the USA, 26 and in South Africa. 27 Organized Health Education and Communication programme has also been very useful in bolstering other health behaviours including HIV risk perceptions among in-school adolescents; 28 Diabetes- knowledge of community dwelling adults; 29 as well as health and food safety knowledge, attitude, and practice among food handlers during the COVID-19 pandemic. 30 This was why there was a strong recommendation for effective health education and communication to encourage vaccine acceptance in Nigeria. 7

The study found no significant interaction effect of gender and the willingness to accept COVID-19 vaccines in the pre-test and post-test group. This finding was surprising as females are generally more health conscious than men. 31 Therefore, it is expected that they will be more willing to accept COVID-19 vaccines than do men. This finding is contrary to some early COVID-19 studies that reported lower intentions to accept COVID-19 vaccination among women. 32, 33 Females have also been reported to be more undecided in accepting vaccination. 34 Furthermore, in a meta-analysis involving several countries, significantly fewer women than men showed willingness to accept vaccination than. 35 The low willingness to accept vaccination may be linked to safety concerns relating to COVID-19 vaccines since females are more cautious in taking health-related decisions than males.

There was no significant interaction effect of level of education and the willingness to accept COVID-19 vaccines in the pre-test and post-test group. The finding was surprising since education is positively associated with better understanding of diseases' prevention. Hence, more educated respondents should have higher willingness to accept vaccination before and after MHCI. This finding is in variance with some previous reports. One univariate analysis showed that education level could influence willingness to accept vaccination 36. Specifically, people with higher levels of education (master's degree and above) and those with lower levels of education (high school and below) were significantly more hesitant towards vaccination than those at middle-level (junior college or bachelor's degree) ^37^

Furthermore, no interaction effect of age and willingness to accept COVID-19 vaccines was found in the pre-test and post-test group. This was expected as all the respondents were adults and the difference in their ages is not expected to influence whether or not they would accept the vaccine. This finding is countered in previous study which found younger respondents to be less willing to accept vaccination. 33 The disparity in the finding could be attributed to the rich contents of MHCI used in the present study which enhanced the willingness to accept the vaccine notwithstanding their gender, level of education or age.

## Conclusion and recommendation

Findings from this study have shown the MHCI is effective in promoting the willingness to accept COVID-19 vaccines. The responses elicited prior to the treatment show that the respondent was very unwilling to accept COVID-19 vaccines. This is a microcosm of the Nigerian situation where unwillingness to accept COVID-19 vaccination is rife. This prompted the desire for the present study. After treatment with MHCI, the respondents indicated heightened willingness to accept the vaccine notwithstanding gender, level of education and age. We firmly conclude, therefore, that our MHCI is veritable in bolstering the willingness to accept COVID-19 vaccination. In view of this, we recommend that Federal and State ministries of health should, as a matter of urgency, adopt and support the widespread use of MHCI in schools, communities, parastatals and religious institutions. This will help in upscaling vaccination acceptance and coverage. This way, Nigeria may be able to reach herd immunity in shortest possible time.
